# A Targeted Capture Linkage Map Anchors the Genome of the Schistosomiasis Vector Snail, *Biomphalaria glabrata*

**DOI:** 10.1534/g3.117.041319

**Published:** 2017-05-19

**Authors:** Jacob A. Tennessen, Stephanie R. Bollmann, Michael S. Blouin

**Affiliations:** Department of Integrative Biology, Oregon State University, Corvallis, Oregon 97331

**Keywords:** *Biomphalaria*, linkage map, linkage disequilibrium, mybaits, *Schistosoma*

## Abstract

The aquatic planorbid snail *Biomphalaria glabrata* is one of the most intensively-studied mollusks due to its role in the transmission of schistosomiasis. Its 916 Mb genome has recently been sequenced and annotated, but it remains poorly assembled. Here, we used targeted capture markers to map over 10,000 *B. glabrata* scaffolds in a linkage cross of 94 F1 offspring, generating 24 linkage groups (LGs). We added additional scaffolds to these LGs based on linkage disequilibrium (LD) analysis of targeted capture and whole-genome sequences of 96 unrelated snails. Our final linkage map consists of 18,613 scaffolds comprising 515 Mb, representing 56% of the genome and 75% of genic and nonrepetitive regions. There are 18 large (> 10 Mb) LGs, likely representing the expected 18 haploid chromosomes, and > 50% of the genome has been assigned to LGs of at least 17 Mb. Comparisons with other gastropod genomes reveal patterns of synteny and chromosomal rearrangements. Linkage relationships of key immune-relevant genes may help clarify snail–schistosome interactions. By focusing on linkage among genic and nonrepetitive regions, we have generated a useful resource for associating snail phenotypes with causal genes, even in the absence of a complete genome assembly. A similar approach could potentially improve numerous poorly-assembled genomes in other taxa. This map will facilitate future work on this host of a serious human parasite.

The aquatic planorbid snail *Biomphalaria glabrata* is one of the most notorious mollusk species given its role in the transmission of schistosomiasis, a neglected tropical disease (Bayne 2009). This snail is the primary vector in the New World, and other *Biomphalaria* species are important vectors in Africa and the Middle East. Schistosomiasis in humans and other mammals is caused by infection with adult blood flukes, *Schistosoma*. Schistosomes infect > 200 million people (King *et al.* 2005; Steinmann *et al.* 2006), causing a chronic disease with a global impact of 13–56 million disability-adjusted life years (King 2010). These flatworm parasites require passage through an obligate intermediate host snail to complete their life cycle. Thus, a key strategy to combatting infection could involve blocking transmission at the snail stage.

Due to the widespread interest in this species, the genome of *B. glabrata* has recently been sequenced, assembled, and annotated (Adema *et al.* 2017). In addition to its medical relevance, this effort represented one of the few lophotrochozoan genome projects and it produced one of the most thoroughly-characterized genomes of a gastropod to date, alongside *Aplysia californica* (AplCal3.0; https://www.ncbi.nlm.nih.gov/assembly/GCF_000002075.1/) and *Lottia gigantea* (Simakov *et al.* 2013). The assembled genome *BglaB1* contains 899 Mb of sequence, estimated to be 98% of the complete genome. However, with an N50 of 48 kb, the practical utility of the genome is limited. It is not known how the > 300,000 scaffolds combine to make the 18 haploid *B. glabrata* chromosomes. A genetic marker linked to a trait of interest is likely to occur on a different scaffold than the causal gene underlying the phenotype. Indeed, although there are now several loci associated with resistance to schistosome infection (Knight *et al.* 1999; Goodall *et al.* 2006; Ittiprasert *et al.* 2013; Tennessen *et al.* 2015a,b; Allan *et al.* 2017), the genomic regions surrounding these loci remain incompletely characterized. Thus, the partial assembly presents a challenge for genetic mapping studies hoping to identify causal genes, as well as potential studies of genome evolution and organization. Fortunately, a complete assembly is not necessary to overcome this hurdle. The *B. glabrata* genome is very repetitive (Adema *et al.* 2017), and many scaffolds only contain sequence that is very similar to sequence found elsewhere in the genome. Such repetitive scaffolds are unlikely to contain either functional genes or reliable molecular markers, and assembling them is a low priority. What matters most for practicality is the relative positions of scaffolds containing unique sequence, especially coding genes.

Linkage mapping provides a practical way to categorize the scaffolds of an incomplete genome assembly. In particular, sequence-based markers can be assigned unambiguously to scaffolds, allowing scaffolds to be organized into, and ordered within, LGs (Ott *et al.* 2015). The initial genome project included a linkage map based on RAD tags (Adema *et al.* 2017). However, most scaffolds did not have an informative RAD tag, so only 145 Mb could be assigned to LGs. RAD tags sample the genome randomly, and one cannot control where a marker will occur. In contrast, targeted capture is an increasingly popular technology that allows markers to be placed in particular sections of the genome (Jones and Good 2016). For this reason, targeted capture is a useful tool for linkage mapping (Tennessen *et al.* 2013, 2014; Chevalier *et al.* 2014; Hisano *et al.* 2017). In the study described here, we employed targeted capture to create a substantially improved linkage map for *B. glabrata*. By focusing on large and unique scaffolds, we were able to map most of the functionally relevant regions of the genome.

## Materials and Methods

### Approach

**Figure 1:**
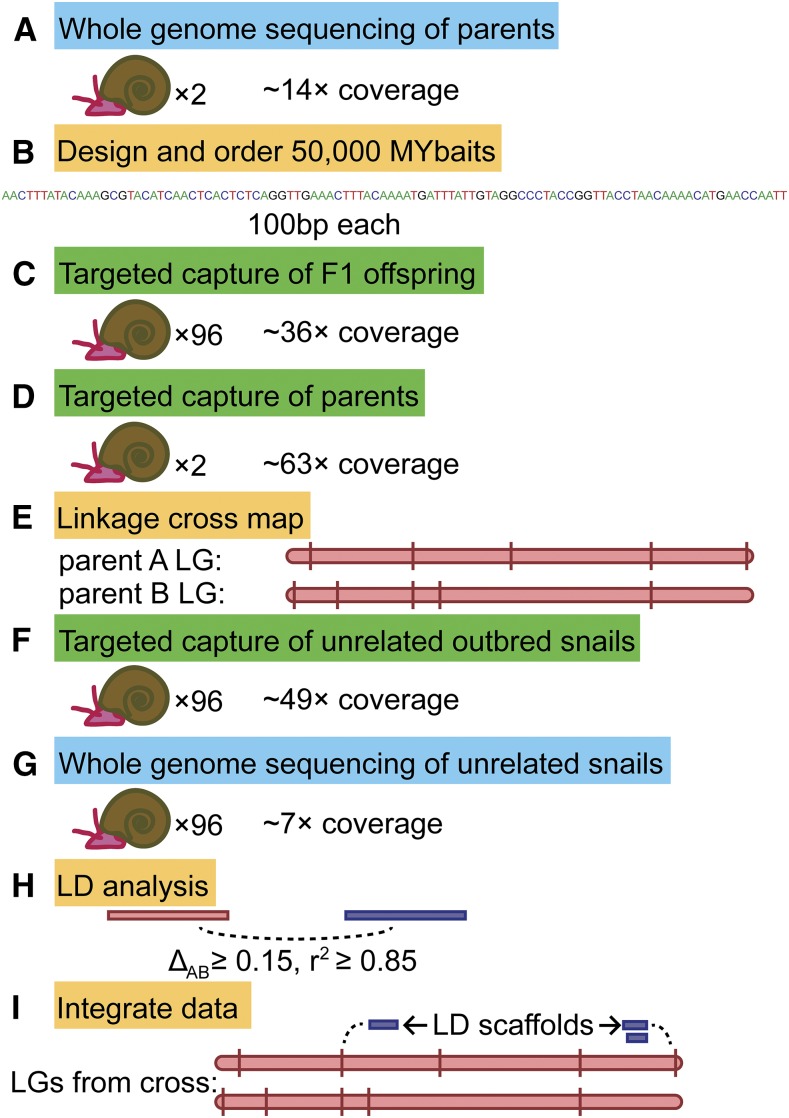
Methodology summary. (A) We sequenced the complete genomes of two unrelated parents, which (B) guided our designing of targeted capture probes, which we used to genotype (C) the F1 offspring and (D) the parents. (E) Segregating targeted capture markers were mapped into LGs. We also performed both (F) targeted capture and (G) whole-genome sequencing on a set of unrelated outbred snails to (H) infer LD. (I) We integrated these data with each other to generate the final LGs. LD, linkage disequilibrium; LGs, linkage groups.

Our overall approach is summarized in Figure 1. In brief, we used a combination of targeted capture and whole-genome sequencing to generate markers on thousands of scaffolds in both a mapping family and a set of unrelated snails. We integrated these data with each other to assign scaffolds to LGs. For the linkage cross, we used a family of two unrelated outbred parents of *B. glabrata* strain 13-16-R1 and 96 of their F1 offspring. This is the same family that was used to generate a preliminary linkage map based on RAD tags, described previously (Bioproject Accession PRJNA288880; Adema *et al.* 2017). The two parents are arbitrarily designated parent A and parent B. Since *B. glabrata* is hermaphroditic, either parent may have served a maternal or paternal role. There are no sex chromosomes or sex linkage other than via mitochondrial DNA, which we ignored. For LD analysis, we randomly selected 96 snails from a set of unrelated outbred 13-16-R1 snails that had been previously genotyped at several candidate loci for parasite resistance (Tennessen *et al.* 2015b). For all samples in this study, DNA was extracted from headfoot tissue with an established CTAB procedure (Goodall *et al.* 2006).

### Genomic evaluation

The feasibility of our approach is based on the observation that a minority of scaffolds in *BglaB1* contain unique and/or genic sequence. To characterize *BglaB1*, we divided it into 600 bp fragments and BLASTed these against *BglaB1* with blastn (version 2.2.31) using an *E*-value threshold of 1e−50 and an identify threshold of 85%, without the dust filter. Fragments with a single hit were considered to be unique.

### Parental genome sequencing and probe design

Our approach to linkage mapping using targeted capture closely followed the methods outlined in Tennessen *et al.* (2013). In order to identify segregating polymorphisms in the linkage cross family, we sequenced the complete genomes of the two parents, each in its own lane of the Illumina HiSeq 2000 at Oregon State University with 100 bp single-end reads. We aligned reads to the *B. glabrata* reference genome *Bgla1B1* (Adema *et al.* 2017) using BWA version 0.6.2 (command bwa aln -n 0.001; Li and Durbin 2009) and converted genotypes to vcf format with SamTools version 1.1 (Li *et al.* 2009). For all informative polymorphisms (heterozygous in one or both parents), we extracted 201 bp flanking segments centered on the polymorphism. We then BLASTed these flanking segments against *BglaB1* using an *E*-value threshold of 1e−50 and an identity threshold of 85%, without the dust filter. Flanking segments that matched only a single scaffold were retained as unique. From each unique flanking segment, we attempted to design a 100 bp probe sequence overlapping the informative polymorphism and with GC content ≥ 30%, at least 97 homozygous sites, no homopolymer runs longer than 5 bp, and no unknown (*N*) bases. These probes were further filtered to retain a maximum of 3 probes per scaffold, except for scaffolds over 100 kb, for which more were retained (three probes in the first half and three in the second half for scaffolds between 100 and 200 kb, and three probes in each third of any scaffold over 200 kb). This round of filtering favored probes that were unique in the genome with an identify threshold of 75%, that were centered on the targeted polymorphism, that overlapped few or no other heterozygous sites, and that occurred near the middle of the scaffold. After generating this set of probes, we identified all scaffolds > 100 kb that were still absent, and we reran the probe design pipeline with lower stringency (GC content ≥ 25%, at least 96 homozygous sites, and no homopolymer runs longer than 8 bp) in order to find probes for these large scaffolds, and added these probes to our list. The resulting probe sequences were sent to MYcroarray (Ann Arbor, MI) for evaluation. Based on their recommendations, we chose a set of 40,000 probes for synthesis as biotinylated RNA MYbaits to be used as targeted capture probes. This set included a single probe for scaffolds < 100 kb, and up to three probes for scaffolds > 100 kb. As MYcroarray was unable to synthesize all of these probes successfully, we then chose an additional set of 10,000 probes for synthesis from the same filtered and evaluated list of candidates. Our probe set included two probes on Scaffold191 which was subsequently found to be a bacterial contaminant and not of snail origin (Adema *et al.* 2017); for all further analyses we have excluded this scaffold.

### Targeted capture and additional genome sequencing

Following our previous approach (Tennessen *et al.* 2013), we target captured sequences for all samples. DNA from the 96 offspring and the 96 unrelated snails was prepped for Illumina sequencing with the TruSeq Nano DNA HT kit with dual indexing, pooled, target-enriched following the MYcroarray protocol, and split among four lanes of the Illumina HiSeq 3000 at Oregon State University with paired-end sequencing and 150 bp reads. In addition to targeted capture, we sequenced the complete genomes of the 96 unrelated snails in eight lanes of the Illumina HiSeq 3000 at Oregon State University with paired-end sequencing and 150 bp reads. In order to ensure high coverage in the parents at linkage map markers, we also performed target capture on the parents and ran these samples on the Illumina MiSeq at Oregon State University with paired-end sequencing and 300 bp reads. We trimmed reads with Trimmomatic version 0.30 (Bolger *et al.* 2014), aligned them to *BglaB1* using BWA version 0.6.2 (command bwa aln -n 0.001; Li and Durbin 2009) and converted genotypes to vcf format with SamTools version 1.1 (Li *et al.* 2009).

### Linkage cross mapping

Following our previous approach (Tennessen *et al.* 2013), we used OneMap version 2.0.4 (Margarido *et al.* 2007) on R version 3.2.3 to assign markers to LGs. We converted the vcf genotypes to OneMap format and filtered them as described below (see *Marker filtering*). Based on segregation patterns and parental genotypes, we categorized all markers according to OneMap designations as D1.10 (parent A heterozygous but not parent B), D2.15 (parent B heterozygous but not parent A), B3.7 (both parents heterozygous), C.8 (both parents heterozygous for null allele), B1.5 (parent A heterozygous, parent B heterozygous for null allele), or B2.6 (parent A heterozygous for null allele, parent B heterozygous). Markers were initially assigned to LGs in OneMap using a LOD threshold of 3.4, rather than the more permissive typical LOD threshold of 3, as we found that this more conservative threshold prevented seemingly spurious associations. Small LGs were manually combined if they shared at least four scaffolds and the merged LG could maintain a consistent scaffold order in both parents. We assumed that putative recombination events observed only at a single site were genotyping errors rather than true recombination events, and thus our estimates of genetic distance (reported in centimorgans) are conservative.

### Integration of additional scaffolds via LD

For the 96 outbred snails, we recovered markers from both targeted capture and whole-genome sequencing. These were filtered as described below (see *Marker filtering*). Targeted capture and whole-genome sequencing variants were then analyzed jointly. For all pairs of variants, we calculated pairwise composite LD as Δ_AB_ and *r*^2^ (χ^2^ divided by sample size), using the Perlymorphism package (Bio::PopGen, BioPerl version 1.007000; Stajich and Hahn 2005). We considered a correlation to be meaningful if Δ_AB_ ≥ 0.15 and *r*^2^ ≥ 0.85. For scaffolds absent from the linkage cross map, we added them to LGs if they showed meaningful LD with at least one variant each on at least two mapped scaffolds on the same LG. If a scaffold met this LD match criteria for multiple LGs, the scaffold was assigned equally to all of them. Any scaffold that ended up assigned to more than two LGs was discarded.

### Marker filtering

For all sets of markers, we performed filtering steps to exclude putative markers that could either be sequencing artifacts or else provide little power toward the goal of finding linkage among scaffolds. For the targeted capture linkage cross dataset, we only retained polymorphisms if they had at least 10 individuals with each of at least two different genotypes, and if 30% or fewer samples had Phred-scaled quality scores under 15, which were scored as missing data. Samples with missing data at > 50% of sites were excluded from subsequent analysis. All polymorphisms passing these criteria were considered as potential linkage map markers, regardless of whether they were targeted by our probes. For the targeted capture LD dataset, we only retained polymorphisms if they had at least 20 individuals with each of at least two different genotypes, and if they were consistent with Hardy–Weinberg equilibrium at α of 10^−3^. As with the linkage cross dataset, we required at least 70% of genotypes to have Phred-scaled quality scores of at least 15, but unlike the linkage cross we included genotypes with Phred-scaled quality scores between 3 and 15 rather than scoring them as missing. In order to reduce the size of the dataset for computational ease, we then removed redundant markers. Specifically, we removed variants that had a very similar genotype profile to other variants on the same scaffold, until all variants on each scaffold differed by at least eight genotypes. For the whole-genome LD dataset, we first performed the same filtering steps as for the targeted capture LD dataset. Then, in order to produce a dataset of manageable size for pairwise LD (*i.e.*,< 10^6^ × 10^6^ pairwise comparisons), we excluded sites that were covered by a targeted capture probe, we only included scaffolds that contained unique sequence as defined by our BLAST analysis, and we only included indel polymorphisms if there were no single-nucleotide polymorphisms on the same scaffold.

### Synteny analysis

In order to examine patterns of synteny, we identified orthologs with *A. californica* and *L. gigantea* (Simakov *et al.* 2013). Although these are the other available annotated gastropod genomes, they are not very closely related to *Biomphalaria*, which shared a common ancestor with *Aplysia* ∼200–250 million years ago (MYA), and with *Lottia* ∼450–500 MYA (Zapata *et al.* 2014). Thus, incongruences in synteny may reflect either real evolutionary rearrangements or assembly errors. We used reciprocal BLAST analysis of the amino acid sequences of all annotated *A. californica* or *L. gigantea* genes *vs.* the amino acid sequences of all annotated *BglaB1* genes. Sequence pairs were retained as orthologs if they were reciprocal best hits at an *E*-value threshold of 1e−100 and both were at least 100 amino acids long. We examined *A. californica* or *L. gigantea* scaffolds that had orthologs on more than one mapped *BglaB1* scaffold, and we measured how often these occurred on the same *B. glabrata* LG.

### Data availability

All Illumina reads have been uploaded to NCBI SRA (Bioproject Accession PRJNA380244). Sequence coverage information for all samples is in Supplemental Material, Table S1. The linkage map results are available as Table S2, Table S3, Table S4, Table S5, and Table S6. Genotypes used in the linkage cross are in Table S2, LD values are in Table S3, linkage map positions of all scaffolds are in Table S4, linkage map positions of all markers are in Table S5, and genotypes used in LD mapping are in Table S6. Regions of exceptionally high LD are highlighted in Table S7. The MYbait sequences are in File S1.

## Results

### Genomic evaluation

We found 39,472 scaffolds with unique sequence, 12% of the total, but since these were biased toward larger scaffolds they comprised 720 kb (79%) of the genome, as well as 98% of the 14,332 predicted genes.

### Parental genome sequencing and probe design

We obtained 193,404,516 and 202,803,587 Illumina reads for parent A and parent B, of which 119,459,945 and 132,435,725 aligned to *BglaB1*, respectively (mean coverage = 14 ×, Table S1). We initially identified 1,871,338 and 2,279,001 sites heterozygous in one or the other parent, as well as 1,403,585 sites heterozygous in both. We extracted 4,334,479 flanking segments of 201 bp to BLAST against *BglaB1*, of which 2,460,129 were unique and matched 80,328 scaffolds. From these, we designed 921,440 probes on 44,315 scaffolds informative in parent A, as well as 1,091,930 probes on 57,905 scaffolds informative in parent B. These were filtered to a set of 217,304 probes. There were 234 scaffolds > 100 kb that were not represented by this set, so we designed additional probes targeting these scaffolds and generated a set of 230,300 probe sequences that we sent to MYcroarray for evaluation. From these, we chose 50,000 probes for synthesis as MYbaits, of which 46,023 were successfully synthesized (File S1). These represented 36,611 scaffolds comprising 685.9 Mb, 91% of unique scaffold sequence, and 96% of all genes.

### Targeted capture and additional genome sequencing

At targeted sites, we obtained a mean coverage of 36 × (median = 36 ×) for the linkage cross samples, 49 × (median = 50 ×) for the LD samples, and 69 × and 57 × for parent A and parent B, respectively (Table S1). After filtering of targeted site data, we identified 144,262 polymorphisms on 17,340 scaffolds for the linkage cross. Two samples had missing data at > 50% of sites and were excluded from subsequent analysis, leaving 94 offspring in the linkage cross (median missing data = 9%). Each offspring was heterozygous for thousands of markers that were homozygous in parent A, and thousands of other markers that were homozygous in parent B, proving that no progeny were products of selfing. We identified 203,878 polymorphisms on 22,294 scaffolds for the LD analysis. For the whole-genome sequencing of the LD samples, we obtained a mean and median coverage of 7 × per sample (Table S1). We initially called 17,437,000 polymorphisms from the whole-genome data. After merging with the targeted capture data and full filtering, there were 349,610 polymorphisms on 26,012 scaffolds for the LD analysis.

### Linkage cross mapping

**Figure 2:**
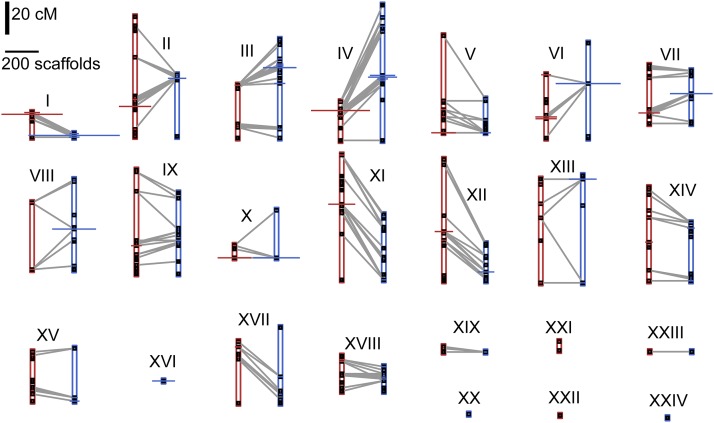
Linkage groups generated by linkage cross. For all 24 linkage groups, the map positions for parent A (red) and/or parent B (blue) are shown as black squares (centimorgan distance along *y*-axis; scale bar in upper left). At each map position, the *x*-axis width of the horizontal bar indicates the number of scaffolds that map to that location (scale bar in upper left; scaffolds subsequently added via linkage disequilibrium not shown). Gray lines indicate where the same scaffold maps in both parents.

From the linkage cross, we initially assigned 49,253 markers on 11,134 scaffolds comprising 412 Mb to 26 LGs based on the 3.4 LOD criterion. In two cases, we merged a small LG containing markers from a single parent with a large LG containing markers from both parents, because the LGs shared at least four scaffolds with markers in different parents. Thus, our final map contains 24 LGs (Figure 2, Table 1, and Table S2). Most (77%) markers were heterozygous in one parent or the other, with 22% heterozygous in both parents, and the remaining 1% of markers showing a more complicated pattern involving a null allele. Due to the large number of markers heterozygous in both parents, it was straightforward to merge the data from both parents into composite LGs. As expected given the 18 haploid snail chromosomes, there were 18 large LGs representing over 9–54 Mb each, while all remaining LGs were under 1.5 Mb. A similar size discrepancy remained after adding additional scaffolds to the map (described below). We hypothesize that the 18 largest LGs each correspond to a different chromosome, and we refer to this set of 18 as the “core” LGs. All but one of the core LGs contain both parent A and parent B markers. In terms of genetic distance, LGs ranged in size from 0.0 to 75.5 cM (parent A) and 0.0–81.9 cM (parent B).

**Table 1:** Linkage groups generated

LG	Parent A cM	Parent B cM	Cross Markers	Scaffolds	Mb	Key Loci
I	14.9	2.1	7,954	2,275	63.410	*prx621**^a^*, *infPhox**^a^*, *Integrin* α-*3 precursor**^b^*, *ferritin**^c^*, *BgGRN**^d^*
II	72.3	37.2	2,582	1,278	44.126	*Coagulation factor XI**^b^*, *BgCREB**^e^*
III	31.9	59.6	3,901	1,267.5	42.570	*tal1–100**^a^*, *Galectin-IIa**^b^*, *p63**^f^*
IV	23.4	81.9	4,154	1,835.5	38.203	*Peptidoglycan recognition protein**^b^*
V	58.5	20.2	2,789	965	36.603	*actin**^c^*, *gpx65**^a^*, *gpx97**^a^*, *prx1**^a^*, *Galectin-IIIa**^b^*, *frep1**^g^*
VI	38.3	57.4	5,020	1,659	33.382	*phox22**^a^*, *Inter* α*-trypsin inhibitor**^b^*, *Cystatin**^b^*, *LBP/BPI**^b^*, β*-1*,*3-glucanase**^b^*
VII	35.1	31.9	3,075	1,127	29.802	*RADres**^h^*, *BgSTAT1**^e^*, *BgSTAT2**^e^*
VIII	40.4	54.3	2,685	1,080	28.083	*frep5**^g^*, *frep7**^g^*, *hcl-2**^i^*
IX	62.8	48.9	1,362	592	26.313	*Guadeloupe Resistance Complex* (including *grctm6*)*^j^*,*^k^*, *BgTLR**^l^*, *spondin-1**^b^*, *sod1**^m^*,*^n^*, *bmplys**^h^*,*^o^*, *prx4**^a^*,*^n^* (Figure 5)
X	7.4	28.7	2,930	1,190.5	24.348	*frep3**^p^*, *frep4**^g^*, *frep11**^g^*, *duox584**^a^*
XI	75.5	39.4	2,168	669.5	22.606	*nox2**^a^*, *BgRelish**^e^*
XII	73.4	22.3	2,144	961	22.396	*hsp70**^c^*, *RAPD QTL**^q^*
XIII	59.6	64.9	2,197	900.5	22.389	*gpx2404**^a^*, *Matrilin**^b^*, *Theromacin**^b^*
XIV	56.4	35.1	1,062	562.5	17.916	*Metalloproteinase**^b^*
XV	29.8	31.9	1,263	449.5	17.663	
XVI	0	0	1,300	621.5	14.697	
XVII	37.2	45.7	894	415	12.792	*Serine protease inhibitor**^b^*
XVIII	21.3	14.9	1,307	540.5	12.782	
XIX	3.2	0	122	94	1.407	
XX	0	0	181	26	1.286	
XI	5.3	0	86	40	1.276	
XII	0	0	11	18.5	0.469	
XIII	0	0	61	33	0.323	
XIV	0	0	5	12	0.210	
Total	746.7	676.4	49,253	18,613	515.053	

LG, linkage group; Parent A cM, genetic distance spanned by parent A markers in centimorgans; Parent B cM, genetic distance spanned by parent B markers in centimorgans; Cross Markers, number of markers used in linkage cross map (LD markers not included); Scaffolds, total number of scaffolds in final LG (some scaffolds are split among LGs, so totals are not integers); Mb, combined size of all scaffolds in final LG in megabases; Key Loci, notable or representative loci found on LG. LD, linkage disequilibrium.

a Larson *et al.* (2014).

b Mitta *et al.* (2005).

c Adema *et al.* (2017).

d Pila *et al.* (2016b).

e Zhang and Coultas (2011).

f Baričević *et al.* (2015).

g Léonard *et al.* (2001).

h Tennessen *et al.* (2015b).

i Peña and Adema (2016).

j Tennessen *et al.* (2015a).

k Allan *et al.* (2017).

l Pila *et al.* (2016a).

m Goodall *et al.* (2006).

n Blouin *et al.* (2013).

o Galinier *et al.* (2013).

p Hannington *et al.* (2012).

q Knight *et al.* (1999).

### Integration of additional scaffolds via LD

We identified 4,615,457 instances of meaningful LD (Δ_AB_ ≥ 0.15, *r*^2^ ≥ 0.85) between two markers on different scaffolds, spanning 21,971 scaffolds. We used these results to add 7479 scaffolds to the map, comprising 103 Mb. The remaining LD scaffolds were either already on the map from the linkage cross, were in LD with scaffolds on > 2 LGs, or else were only in LD with scaffolds which themselves were not on the map. All 1,305,886 scaffold pairs showing meaningful LD are listed in Table S3, providing additional data on which scaffolds are likely to be physically close. The scaffold showing the greatest LD was Scaffold797, in LD with 1788 other scaffolds.

**Figure 3:**
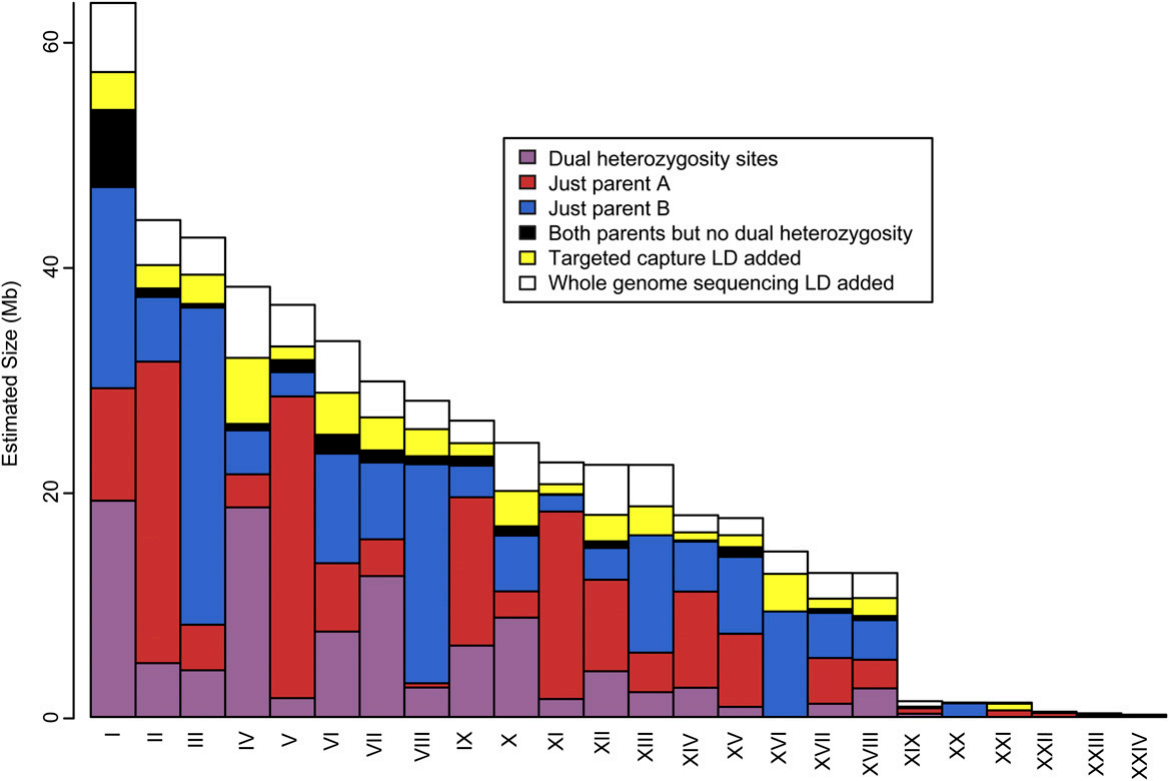
Physical sizes of LGs. The cumulative size of all scaffolds on all LGs is shown in megabases. Colors indicate how scaffolds were assigned: via markers heterozygous in both parents (purple), just parent A (red), just parent B (blue), markers heterozygous in either parent but never in both (black), LD from targeted capture (yellow), or LD from whole-genome sequencing (white). The core linkage map consisting of the first 18 LGs represents the vast majority of mapped scaffolds. LD, linkage disequilibrium; LGs, linkage groups.

**Figure 4:**
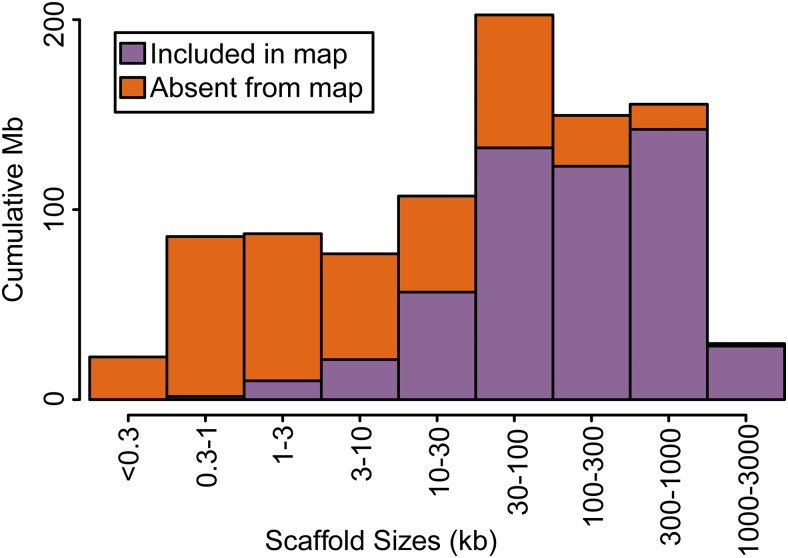
Sizes of scaffolds in *BglaB1* and in the linkage map. Scaffolds are binned by size. Smaller scaffolds are less likely to have been mapped, but most of the genome is found on scaffolds > 30 kb and most of this sequence has been mapped. Thus, 56% of the genome has been assigned to linkage groups, including 75% of the genic and nonrepetitive regions.

**Figure 5:**
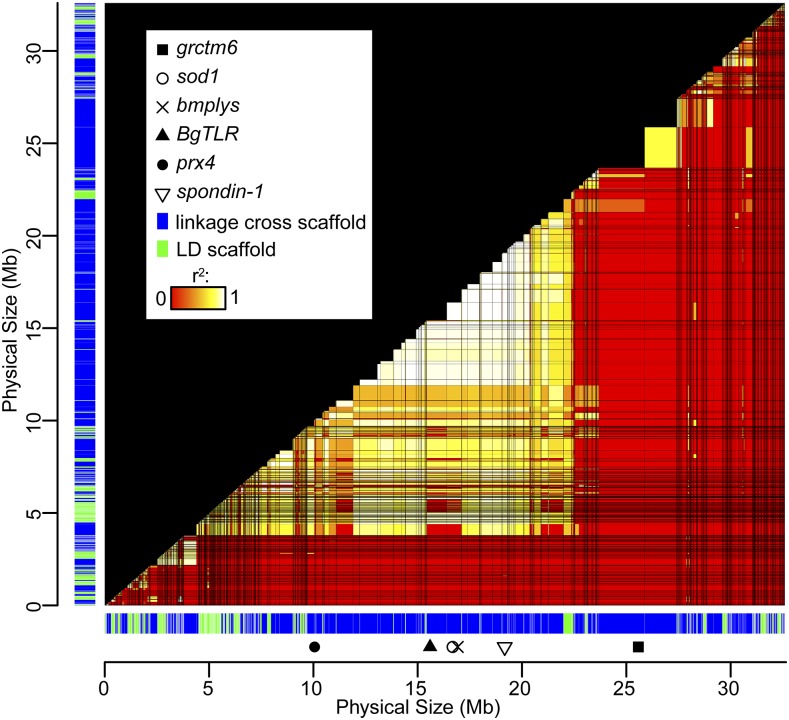
LD across LG IX. All scaffolds are shown in mapped order along both the *x*- and *y*-axes. Scaffold color indicates whether placed in the linkage cross (blue) or by LD (green). Scaffolds are scaled relative to physical size; scaffolds shared with other LGs have been reduced in size accordingly. An arbitrary spacer of 10 kb is inserted between all scaffolds. For each scaffold pair, the highest observed pairwise *r*^2^, for which Δ_AB_ ≥ 0.15, is plotted among all scaffolds using heat map colors (red: no Δ_AB_ ≥ 0.15 and/or *r*^2^ = 0; white: *r*^2^ = 1). Several notable immune-relevant loci occur on this LG, some of which are indicated. A large block of high LD stretching for several megabases (Table S7) includes the immune-relevant genes *spondin-1*, *sod1*, *bmplys*, and *BgTLR* (Mitta *et al.* 2005; Goodall *et al.* 2006; Galinier *et al.* 2013; Pila *et al.* 2016a). The linkage between *prx4* and *sod1* has been previously examined (Blouin *et al.* 2013). The Guadeloupe Resistance Complex contains several potential immunity genes, of which *grctm6* is the most extensively studied candidate (Tennessen *et al.* 2015a; Allan *et al.* 2017). LD, linkage disequilibrium; LG, linkage group.

The final map consists of 18,613 scaffolds comprising 515 Mb (Figure 3, Table 1, and Table S4). These scaffolds represent a majority (56%) of the genome, and larger scaffolds were more likely to be included (Figure 4). The core linkage map alone contains 510 Mb, which is 55.7% of the genome. A total of 73,851 segregating markers contributed to the map, of which 67% were from the linkage cross, 6% were from targeted capture LD, and 27% were from whole-genome sequencing LD (Table S5 and Table S6). We arranged LGs by size in megabases in descending order, designated by Roman numerals. Thus, the largest LG is I and the smallest LG is XXIV. These designations bear no relationship to the RAD-based LGs that were used to name scaffolds in *BglaB1*. Thus, for example, the full name of Scaffold378 in *BglaB1* is LG17_random_Scaffold378 because it occurs on RAD-based LG 17, but this has nothing to do with LG XVII presented here, and Scaffold378 occurs on LG XIV. The 18 core LGs (I–XVIII) are all over 12 Mb, while the remaining six LGs are all under 1.5 Mb. All core LGs are < 70% the size of the next largest LG. In contrast, LG XIX is only 11% the size of LG XVIII. The fact that this large size discrepancy happens right after the expected number of haploid chromosomes supports the hypothesis that the 18 core LGs represent the 18 chromosomes. Scaffold order within LGs reflects map position in centimorgans as well as LD among scaffolds (Figure 5).

LGs showed notable heterogeneity in relative recombination rate, and occasionally in scaffold order between parents (Figure 2), possibly reflecting inversions or other chromosomal rearrangements. To search for putative suppressors of recombination such as inversions, we identified haplotype blocks consisting of sets of scaffolds with perfect LD (Δ_AB_ ≥ 0.15, *r*^2^ = 1) for at least one marker in all pairwise comparisons. Five of these blocks were of large estimated size (4–6 Mb; Table S7), and all of these corresponded to linkage map positions where, in each parent, > 150 scaffolds comprising over 5 Mb were clustered (Table S7). Thus, the regions of highest LD represent regions of unusually low recombination in both parents, including the largest region of no recombination on LG I (Figure 2). Inversions heterozygous in both parents could suppress recombination without contributing to discrepancies between the parents, which may be due to other inversions or recombination modifiers.

### Synteny analysis

**Figure 6:**
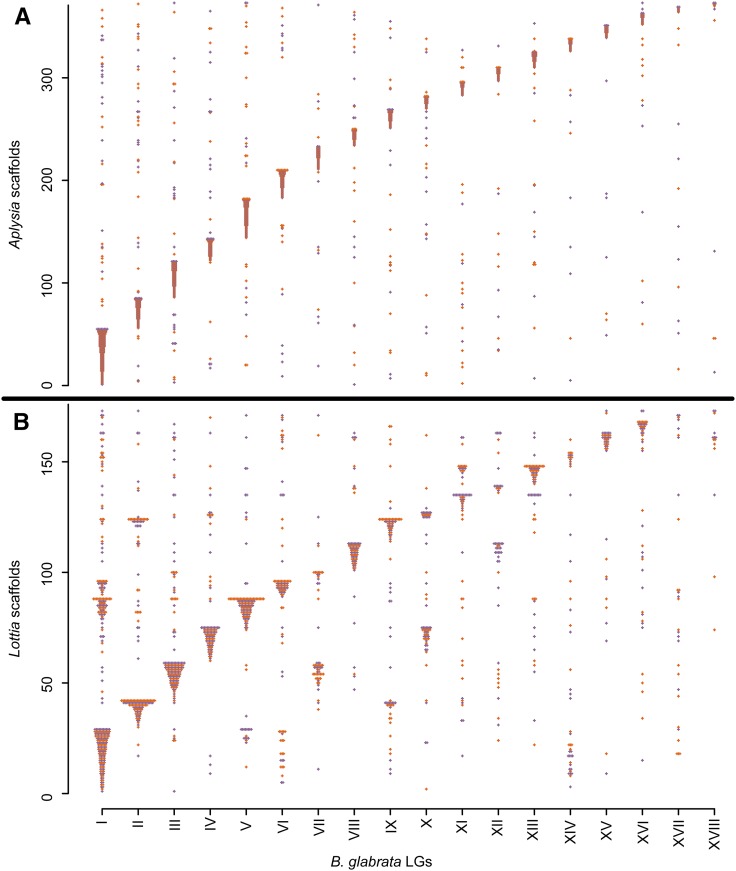
Synteny comparisons with other gastropods. Scaffolds from *A. californica* (A) and *L. gigantea* (B) are plotted along the *y*-axis (scale = count of scaffolds). Each ortholog to a mapped *B. glabrata* gene is plotted along the *x*-axis, in the column corresponding to the *B. glabrata* core LG. Colors alternate for ease of visualization. Most scaffolds match a single *B. glabrata* core LG that contains the majority of orthologs. Unlike *A. californica*, the more distantly-related *L. gigantea* has several scaffolds that tend to be shared between the same two *B. glabrata* LGs, likely indicating chromosomal rearrangements. LGs, linkage groups.

We first examined synteny with *A. californica*, the only other heterobranch with an annotated genome (Zapata *et al.* 2014). We identified 2426 reciprocal best hit orthologs between *B. glabrata* and *A. californica*. From these we identified 373 *A. californica* scaffolds with orthologs on at least two different *B. glabrata* scaffolds. These orthologs occurred on 772 *B. glabrata* scaffolds. For most (87%) of these *A. californica* scaffolds, at least 50% of their orthologous *B. glabrata* scaffolds occurred on the same LG (Figure 6A). Of *B. glabrata* scaffold pairs sharing the same orthologous *A. californica* scaffold, 44% also shared the same LG, 3.7 times more than would be expected by chance. The proportion of *B. glabrata* scaffolds occurring on the most common LG for their *A. californica* scaffold was 82% for scaffolds mapped in the linkage cross and 79% for scaffolds mapped with LD.

We also examined synteny with *L. gigantea*, a more distantly related gastropod. We found an enrichment of synteny but to a lesser degree than with *A. californica*, likely reflecting the greater evolutionary distance. We identified 2509 reciprocal best hit orthologs between *B. glabrata* and *L. gigantea*. From these we identified 173 *L. gigantea* scaffolds with orthologs on at least two different *B. glabrata* scaffolds. These orthologs occurred on 936 *B. glabrata* scaffolds. For most (70%) of these *L. gigantea* scaffolds, at least 50% of their orthologous *B. glabrata* scaffolds occurred on the same LG (Figure 6B). Of *B. glabrata* scaffold pairs sharing the same orthologous *L. gigantea* scaffold, 33% also shared the same LG, 2.7 times more than would be expected by chance. The proportion of *B. glabrata* scaffolds occurring on the most common LG for their *L. gigantea* scaffold was 60% for scaffolds mapped in the linkage cross and 50% for scaffolds mapped with LD. One striking pattern that was not observed in *A. californica* was that many *L. gigantea* scaffolds were nearly evenly split between two *B. glabrata* LGs, and the same pairs of *B. glabrata* LGs tended to share *L. gigantea* scaffolds (Figure 6B). Notable LG pairs included I/V, III/VII, and IV/X. Presumably these do not represent linkage map errors or they would have been observed in the *A. californica* comparison, nor is it clear how errors in the *L. gigantea* assembly could cause this pattern. Thus, they likely represent true large chromosomal rearrangements.

## Discussion

We have generated a dense linkage map for *B. glabrata* containing 18,613 scaffolds. These scaffolds comprise 56% of the genome, 75% of unique sequence, and 75% of genes. We recovered 18 large (> 10 Mb) LGs, matching the expected number of haploid chromosomes. We have not generated a new assembly, because our mapped scaffolds are presumably interspersed with the numerous additional scaffolds that are mostly small, repetitive, and nongenic. Nevertheless, our linkage map serves as a useful resource for associating molecular markers with nearby genes. We have achieved a linkage map N50 of 17.663 Mb. That is, more than half of the genome can be found on the 15 largest LGs, which are all over 17 Mb.

Our approach could potentially improve the numerous eukaryote genomes that have been sequenced but remain only partially assembled due to complex repetitive structures (Ellegren 2014). In particular, two aspects of our methodology are rarely used in genome projects. First, by targeting unique and genic scaffolds, we could assign the majority of the most interesting genomic regions to LGs. Such selectivity is a major advantage of targeted capture over markers with random genomic positions such as RAD tags (Jones and Good 2016). Of course, as whole-genome sequencing becomes more affordable, it may overtake targeted capture in practicality. Second, LD analysis allowed us to assign additional scaffolds to LGs and provides information about the relative order of scaffolds with the same map positon (Figure 5). Yet LD data and linkage cross data are rarely incorporated in the same mapping study.

Despite these improvements, the assembly of unique *B. glabrata* sequence into pseudochromosomes is still far from complete. Although we set out to map the 39,473 scaffolds with unique sequence, fewer than half of these are included in the final assembly. Of course, the remaining unmapped scaffolds are mostly small and constitute a minority of unique sequence, but future efforts could target them. In addition, there were 1251 scaffolds (7%) that mapped to two different LGs. The vast majority of these multi-mapping scaffolds (90%) did not have markers in the linkage cross, and were added via LD. We cannot ascertain whether these represent errors in the existing assembly or spurious associations in our analysis. If they represent assembly errors, it suggests the caveat that scaffolds assigned to an LG via only one or a few markers do not necessarily pertain fully to that chromosome. We have retained these scaffolds in the map as their multiple placement still represents useful information. However, a more conservative map that excludes them would still comprise 466 Mb, or 51% of the genome. Finally, our relatively small mapping population provided limited power for ordering and orienting scaffolds within LGs, and thus most map positions are represented by several scaffolds of unknown relative placement. However, LD results provide an additional guide for inferring the proximity of scaffolds (Figure 5 and Table S3). It is likely that there are at least a few segregating chromosomal rearrangements in this widespread and genetically diverse species, and thus there is no single correct scaffold order. Indeed, the 13-16-R1 population is a hybrid of several strains originating across the Caribbean and Brazil (Newton 1955), and could be polymorphic for such chromosomal rearrangements including inversions or translocations, which may explain some discrepancies between parent A and parent B (Figure 2). All of these areas for improvement could be addressed with additional linkage mapping or by new sequencing technologies (Belton *et al.* 2012; Rhoads and Au 2015).

Assuming we have observed all recombination events, our results suggest an average genome-wide recombination rate of 0.8 cM/Mb. This rate is somewhat low but within the observed range for invertebrates (Wilfert *et al.* 2007), and our conservative approach may undercount recombination events. Some regions of low recombination, especially those corresponding to large blocks of high LD (Table S7), could be due to inversions. If we have missed many recombination events that occur outside our LGs, the actual genome-wide mean rate may be substantially higher. As nearly half of the genome remains unmapped, we cannot compare physical distances with genetic distances in a highly accurate manner. Thus, we have not attempted to estimate recombination rate variation among genomic regions.

Comparisons with the *A. californica* and *L. gigantea* genomes show an enrichment for synteny but also plenty of exceptions from perfect synteny (Figure 6). Some of these exceptions may represent assembly errors, but many likely represent real chromosomal rearrangements that have taken place over gastropod evolution. (Zapata *et al.* 2014). Only with the more distantly-related *L. gigantea* do we repeatedly observe scaffolds split between the same two LGs, suggesting large-scale rearrangements. However, smaller translocations may have reduced synteny with both *A. californica* and *L. gigantea*. Scaffolds mapped with LD show slightly less synteny than scaffolds mapped in the linkage cross, which may reflect a moderately higher error rate in the LD mapping.

The map locations of noteworthy genes illuminate genome organization and provide benchmarks for associating LGs with chromosomes. The *B. glabrata* loci *ferritin*, *actin*, and *hsp70* have been localized to three separate chromosomes via fluorescence *in situ* hybridization (FISH) (Adema *et al.* 2017). Here we show that *ferritin* occurs on LG I, *actin* occurs on LG V, and *hsp70* occurs on LG XII (Table 1). Additional probes with FISH could be used to unite all LGs with chromosome karyotypes. Previous work has shown that several key immunity genes are physically linked, including *sod1*, *prx4*, *cat*, and *bmplys* (Blouin *et al.* 2013, Tennessen *et al.* 2015b). This cluster occurs on LG IX, position 17.0 cM (parent A) or position 20.2 cM (parent B) and overlaps one of the largest blocks of high LD in the genome (Figure 5, Table 1, and Table S7), perhaps harboring an inversion. The Guadeloupe Resistance Complex strongly affects resistance to schistosome infection (Tennessen *et al.* 2015a) and is also found at this map location, though it does not show meaningful LD with the other loci (Figure 5 and Table 1). Other snail genes with major immune functions, including *BgTLR* (Pila *et al.* 2016a) and *spondin-1* (Mitta *et al.* 2005), also map to this LG. However, LG IX is not the only immune-relevant LG, as many important loci tied to schistosome resistance, such as *FREP3* (Hanington *et al.* 2012) and *RADres1* (Tennessen *et al.* 2015b), occur on other LGs (Table 1). Still, haplotypes of LG IX, harboring distinct suites of alleles across many or all of these loci, are likely to have substantially different effects on host–parasite interactions and epidemiological outcomes.

This linkage map will serve as a useful resource in several ways. First, to allow snail geneticists to connect markers for phenotypic traits to causal genes. This goal is particularly important for *B. glabrata*, which is intensively studied in the applied context of schistosomiasis control. Second, to allow for evolutionary studies such as the synteny comparisons reported here as well as other analyses. For example, one can now look for population genetic signatures of natural selection that span multiple scaffolds. Third, to guide further sequencing efforts. Whole-genome sequencing at high coverage using long read technology such as PacBio (Rhoads and Au 2015) is now feasible. An improved genome sequence of *B. glabrata* using these new technologies is likely in the near future. This map will anchor future assemblies and maximize their accuracy.

## Supplementary Material

Supplemental material is available online at www.g3journal.org/lookup/suppl/doi:10.1534/g3.117.041319/-/DC1.
